# Recent Advances in Molten-Carbonate Membranes for Carbon Dioxide Separation: Focus on Material Selection, Geometry, and Surface Modification

**DOI:** 10.1155/2021/1876875

**Published:** 2021-10-29

**Authors:** Shabana Afzal, Atif Khan

**Affiliations:** ^1^Department of Chemistry, MNS-University of Engineering and Technology, Multan, Pakistan; ^2^Department of Chemical Engineering, University of Engineering and Technology, Lahore, Pakistan

## Abstract

Membranes for carbon dioxide permeation have been recognized as potential candidates for CO_2_ separation technology, particularly in the energy sector. Supported molten-salt membranes provide ionic routes to facilitate carbon dioxide transport across the membrane, permit the use of membrane at higher temperature, and offer selectivity based on ionic affinity of targeted compound. In this review, molten-carbonate ceramic membranes have been evaluated for CO_2_ separation. Various research studies regarding mechanisms of permeation, properties of molten salt, significance of material selection, geometry of support materials, and surface modifications have been assessed with reference to membrane stabilities and operational flux rates. In addition, the outcomes of permeation experiments, stability tests, selection of the compatible materials, and the role of interfacial reactions for membrane degradation have also been discussed. At the end, major challenges and possible solutions are highlighted along with future recommendations for fabricating efficient carbon dioxide separation membranes.

## 1. Introduction

Carbon dioxide emission from industrial flue gases and fossil fuels consumption is increasing chronologically, which has become a major cause of global warming. Impact of global warming on climate change is one of the serious threats to human survival. In this regard, the first step is to reduce the CO_2_ emissions by separating it from industrial flue gases and air [1]. CO_2_ separation process should be energetically and economically viable and the separated CO_2_ should be used as a potential carbon source in different chemical processes and fuel production [2]. Currently, in coal fired power plants, ethanolamines are being used for capturing CO_2_ using chemical absorption. This process is cost-effective but production of N-nitrosamines, alkanolamines, and ketones is one of the major disadvantages because of their toxicity, carcinogenic nature, and omnipresence in the environment [3–5]. Similarly, oceanic storage and mineralization are two other prominent methods for CO_2_ capture. Therefore, to limit the amount of CO_2_ and avoid the harmful by-products, carbon dioxide separation is required by an alternative path which should be of low cost, efficient, and robust with low energy consumption [6].

Separation through membranes is one of the feasible approaches which could meet the above requirements. Membrane-based separations can be cost-effective in terms of operational time durations, temperature, pressure, and energy requirements as compared to separations based upon absorption/adsorption processes [7]. Possible membrane materials include polymers [8], inorganic materials [9], metal organic frameworks, and mixed-matrix membranes [10]. The major challenges for the membrane process are high permeability, selectivity, longer operational hours, and stability at high temperature. Organic polymeric membranes are not suitable for carbon dioxide removal at high temperatures in post-/precombustion industrial processes. Microporous inorganic membranes are stable at high temperatures but their performance is decreased due to transport mechanisms such as sintering and molecular sieving at elevated temperatures [11]. Among different classes of membranes reported, one class of membranes suitable for above-mentioned challenges is the ceramic molten-carbonate (MC) dual-phase membrane [12]. These membranes consist of molten-carbonate phase held within the pore structure of inorganic ceramic support. The ceramic support consists of pure oxygen ionic or mixed ionic-electronic conductor ceramic phase. Ceramic-MC dual-phase membranes have been reported as one of the most potential candidates for CO_2_ separation because their permeation flux reaches up to 2.05 ml (STP) cm^−2^·min^−1^, greatly approaching the level of commercial use, and they can be coupled with many industrial reaction systems [13]. However, fabrication of MC membranes is still at its infancy stage and limited to lab scale because of some critical issues which hinder their complete utilization at large scale such as decreased stability at long operational hours, low efficiency, incompatibility to couple with industrial processes, membrane degradation, high cost, and complex fabrication techniques including optimization of ceramic supports.

This review elaborates MC membranes in view of general concept, fundamental mechanisms, components of MC membrane, fabrication methods, and the factors that influence membrane performance. Major challenges that are serving as huge obstacle for membrane performance, stability, and commercialization have also been highlighted along with prospective solutions and recommendations. The membrane fabrication techniques proposed will be the proof-of-concept work for enhanced CO_2_ selectivity and high temperature stability for longer operational hours.

## 2. Membrane Gas-Separation Mechanism

**Figure 1:**
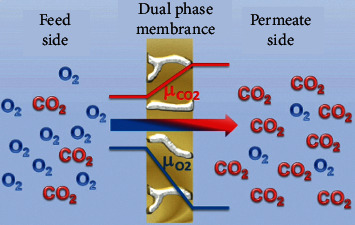
Membrane separation mechanism based on partial pressure difference across the membrane. Pressure difference (driving force), membrane thickness, and surface area are key parameters for membrane performance [14].

The classic molten-carbonate dual-phase membrane consists of two phases. One is the molten-carbonate (MC) phase derived from mixture of carbonate salts. The second is the gaseous phase of carbon dioxide (Figure 1). Difference in partial pressures of CO_2_ at the feed (*P*_*f*_) and permeate boundaries (*P*_*p*_) creates a concentration gradient of carbonate ions in MC phase. When carbonate ions arrive at permeate side, CO_2_ is regenerated by reverse reaction. Membranes separate selective components from a mixture with fast permeant transport. Pressure gradient across the membrane is the driving force for separating target component (Figure 1) [14]. Normally pressure at the feed side (*P*_*f*_) should be greater than pressure at the permeate side (*P*_*P*_). Membrane performance is generally determined by permeation flux (*Ji*), which is defined as the volume of gas *i* passing through the membrane per unit area per unit time at given conditions of temperature and pressure. Permeation flux is measured in SI units, that is, m^3^·m^−2^·s^−1^, or other units, such as ml·cm^−2^·min^−1^, and can be represented by the following equation [15]:

(1) $$\begin{array}{c} Ji=\frac{Ci}{Csg}\times \frac{Q}{\;S}, \end{array}$$

where *C*_*i*_ and *Csg* are the concentrations of component *i* and sweep gas, respectively, measured through gas chromatography (GC). *S* is the effective surface area of membrane and *Q* is the flow rate of sweep gas. However, *Ji* is a function of intrinsic properties of membrane (thickness, materials, and geometry) and operating conditions as well (feed gas concentration, temperature, and pressure). To ensure fair comparison, permeability and permeance are the two better parameters to evaluate membrane performance, as they depend upon intrinsic properties of membranes only. Permeability and permeance are defined as in equations (2) and (4) [16, 17]:

(2) $$\begin{array}{c} \text{permeability}=\frac{Ji}{\Delta P}\times L, \end{array}$$

(3) $$\begin{array}{c} Ji=\text{permeability}\times \frac{\Delta P}{L}, \end{array}$$

(4) $$\begin{array}{c} \text{permeance}=\frac{Ji}{\Delta P}, \end{array}$$

where Δ*P* = *P*_*f*_ − *P*_*p*_ and *L* = membrane thickness.

**Figure 2:**
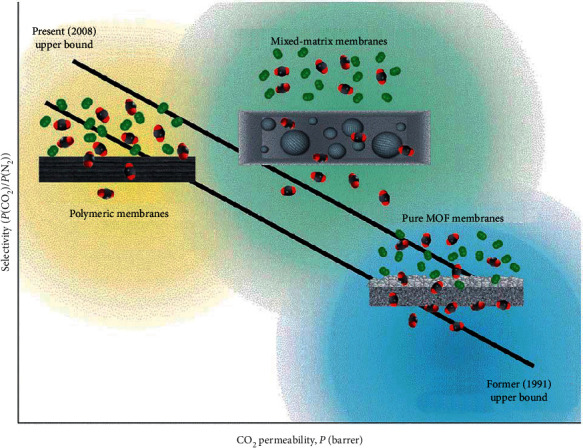
Comparison between selectivity and permeability for CO_2_ for polymeric, MOF, and mixed matrix (both polymeric and MOF) membranes [6, 12].

Selectivity is highly influenced by permeability. In traditional polymeric membranes, driving force depends upon diffusivity and solubility coefficient of the permeant. Diffusion rates with lower resistance pathways can be increased by introducing free pore volume but the selectivity is compromised. The selectivity and permeability relationship can be characterized by Robeson upper bound (Figure 2) [6]. One way to overcome the upper bound without the decrease in selectivity is introducing careers on porous membrane surface. Careers should have high affinity for permeant.

Permeabilities required for economically feasible CO_2_ separation range from 10^−13^ to 10^−12^ mol·m^−1^·s^−1^·Pa^−1^ with carbon dioxide/nitrogen selectivity of 50–100 [18]. Polymeric membranes cannot meet the criteria because of the upper bound described in Figure 2. Zeolite membranes have high selectivity with moderate permeability rates, while metal organic framework (MOF) shows opposite behaviour to zeolite membranes (i.e., high permeability and low selectivity) [19]. However, ceramic supported molten-salt membranes have higher permeability of 10^−12^ to 10^−10^ mol·m^−1^·s^−1^·Pa^−1^ at 600°C and their intrinsic properties show higher selectivity for CO_2_ [20]. Furthermore, these membranes are operational at high temperature range (400–1000°C) which is an extra advantage for their commercial application, that is, CO_2_ removal from hot flue gas streams [20, 21].

**Figure 3:**
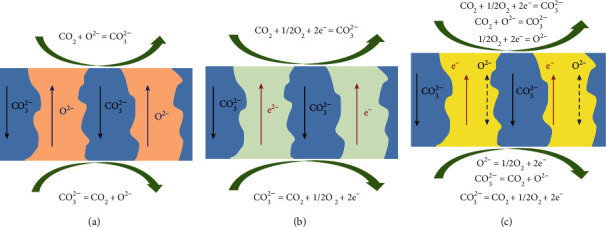
CO_2_ transport mechanisms: (a) MOCC, (b) MECC, and (c) MEOCC [16].

Based upon the mode of CO_2_ transport and reacting species, molten-carbonate (MC) membranes can be classified into three types: (a) mixed oxygen and carbonate ion conducting membranes (MOCC), (b) mixed electron and carbonate ion conducting membranes (MECC), and (c) mixed electron, oxygen, and carbonate ion conducting membranes (MEOCC) [16]. In MOCC membranes, gaseous CO_2_ reacts with oxygen anions (O^2−^) to form CO_3_^2−^ions (Figure 3(a)). These CO_3_^2−^ ions travel through MC phase in the membrane and arrive at permeate side and then CO_2_ is reformed. In MECC membranes, CO_2_ reacts with oxygen atoms in the presence of electrons on an electron conducting porous support (Figure 3(b)). MEOCC membranes exhibit both mixed electron and carbonate ion conduction (Figure 3(c)).

## 3. Molten-Carbonate Membrane Components

Molten-carbonate membrane includes highly ceramic porous support with infiltration of molten salt in pore space. The physical properties of the support such as wettability, thickness, size, volume of pores, and tortuosity have high impact on membrane performance and stability at high temperatures [22]. Supports also facilitate CO_2_ diffusion through membranes by carbonates formation through reaction between CO_2_ and ions in molten salt. Therefore, properties of membrane support can be tuned for increasing CO_2_ permeance. In addition, with the help of molten salts, physical properties of supports such as wettability and vapor pressure can also be modified to meet the requirement at high temperature range [23]. Therefore, it is essential to review the fundamental role and properties of different components of membranes. This section elaborates the various components of molten-carbonate membrane, in context to properties of molten salt, as well as materials and geometries of membranes.

### 3.1. Molten-Carbonate Salts

The molten-carbonate salt commonly used in molten-carbonate membranes is a ternary eutectic carbonate mixture of Li_2_CO_3_-Na_2_CO_3_-K_2_CO_3_ with molar ratio of 43.5 : 31.5 : 25 [12, 24, 25]. Binary eutectic carbonates have also been applied to membranes such as (Li-Na)_2_CO_3_, (Li-K)_2_ CO_3_, and (Na-K)_2_CO_3_ salts with molar ratios of 52 : 48, 62 : 38, and 41 : 59, respectively [26–28]. The purpose of using mixtures of carbonates is to decrease melting point. Melting points of some eutectic binary and ternary carbonates are enlisted in Table 1.

CO_2_ flux strongly depends upon ionic conductivity of molten-carbonate salts infiltrated in ceramic supports. Therefore, proper wetting of molten carbonates in ceramic supports is essential, which in turn is related to thermophysical properties of the molten salt such as viscosity, density, and surface tension. Thermophysical properties depend upon the temperature and environmental conditions around molten salt. Usually, viscosity of the molten salt decreases with increasing temperature. At 650°C, viscosity of a ternary molten carbonate (8 m·Pa·S) becomes comparable to that of water at 20°C (1.02 m·Pa·S) [30, 31]. High temperature is beneficial as low viscosity assists the proper infiltration of molten salt into the pores of ceramic support.

Surface tension of eutectic molten-carbonate mixture (220 mJ·m^−2^) is much lower than that of ceramic supports such as alumina (1.84 J·m^−2^) and yttria-stabilized zirconia, YSZ (1.53 J·m^−2^), at 650°C, leading to very low contact angles between infiltrated carbonates and support [32, 33]. Thus, wetting of supports by carbonates is improved further due to reduced surface tension of carbonates at high temperatures.

### 3.2. Ceramic Supports

Chung et al. pioneered to apply stainless steel as an electron conducting porous support to develop the initial molten-carbonate membrane (MECC) [12]. The conductivity of carbonates decreases at high temperatures because of interfacial reactions between steel and Li_2_CO_3_ leading to formation of LiFeO_2_. Ag has been used as an electron carbonate conducting support. Ag offers high electron conductivity as compared to steel, along with reduction in interfacial reactions at high temperatures [27]. Metal oxides such as Al_2_O_3_, NiO, and ZrO_2_ have also been applied to avoid sintering of Ag and other interfacial reactions [34–36].

**Figure 4:**
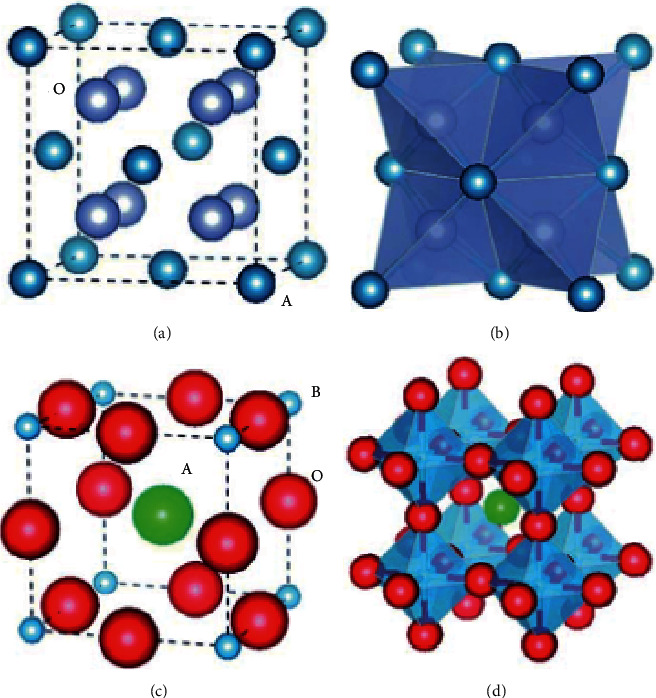
Packing and structural arrangement of (a, b) fluorite structure (AO_2_); blue atoms: A, grey atoms: O, perovskite structure (ABO_3_); green atoms: A, blue atoms: B, red atoms: O [37].

Extensively investigated oxygen ion conducting supports (MOCC), such as samarium doped ceria (SDC) and yttria-stabilized zirconia (YSZ), show good performance at high temperatures. SDC and YSZ exhibit fluorite type structure with AO_2_ formula (Figures 4(a) and 4(b)). Type A cations (Ce, Zr) occupy the face-centered cubic positions, while O^2−^ anions fit into tetrahedral interstices [37]. To enhance oxygen ion conductivity (Figure 4(a)), oxygen vacancy concentration can be increased by doping bivalent and trivalent cations into the parent structure. Based on atomistic simulation results, scandium oxide-doped zirconia has higher oxygen ion conductivity than YSZ and calcium oxide-doped zirconia due to lower energy requirements for dopant solution [39]. Oxygen ion conductivity for rare Earth-doped ceria is reported to be 10^−2^ S/cm. The inclusion of trivalent dopant decreases the activation energy for oxygen ion conduction due to large ionic radius, which results in improving oxygen ion transport [40]. Electronic conductivity is also increased when cerium ion (Ce^+4^) is converted into Ce^+3^ in reducing atmospheres or low oxygen partial pressure [37]. At high temperatures (800°C), cubic fluorite and bismuth oxide (Bi_2_O_3_) show excellent performance for oxygen ion conductivity of 2.3 S/cm^−1^ [41]. If Bi_2_O_3_ is doped with rare Earth cations like Bi_1.5_Y_1.3_Sm_0.2_O_3_ (BYS), then fluorite structure is further stabilized, and oxygen ion conduction is improved at low temperature ranges [42]. However, at elevated temperatures, BYS wettability for carbonates decreases, which would then require some modification in pore size and membrane surface [43]. In this regard, alumina deposition on BYS improves the wettability for molten salts. Similarly, zirconia atomic layer deposition (ALD) also improves the wettability and enhances oxygen and electron ion conduction for longer operational hours [44].

For mixed electron, oxygen, and carbonate ion conduction (MEOCC), materials such as perovskites have been used as supports for molten-carbonate membranes. Figures 4(c) and 4(d) represent a typical perovskite structure ABO_3_, where site A can be a rare Earth metal (La, Ce, or Gd) or alkaline Earth metal (Ba or Sr). Site B can be a transition metal (Mn, Fe, Co, or Cr) or a nontransition metal (Al or Ga) [37]. Perovskite structure consists of BO_6_ octahedrons at corners, where type A cations occupy the cavities formed between octahedrons. To improve thermochemical stabilities at higher temperatures, different perovskite materials have been synthesized and tested. La_0.85_Ce_0.1_Ga_0.3_Fe_0.65_Al_0.05_O_3-*δ*_ (LCGFA) shows good stability at higher temperatures [25]. Similarly, in CO_2_-rich atmosphere, SrFe_0.8_Nb_0.2_O_3-*δ*_ membranes show good performance for 200 operational hours with high chemical stability and thermal cycling [45]. Another perovskite material, Sm_0.6_Sr_0.4_Al_0.3_Fe_0.7_O_3_ composite with fluorite Ce_0.85_Sm_0.15_O_2_ (SDC), is reported to have excellent thermochemical stability at high temperature range (800–950°C) for oxygen ion conduction [46].

### 3.3. Ceramic-Support Geometry

CO_2_ flux in molten-carbonate membranes not only depends on type of materials used in ceramic supports but also is influenced by physical properties of supports such as three-dimensional geometry, thickness, porosity, and tortuosity of the membrane. Therefore, it is essential to review various physical properties followed by corresponding membrane fabrication methods and impact of these properties on membrane performance.

#### 3.3.1. Symmetry and Thickness

**Figure 5:**
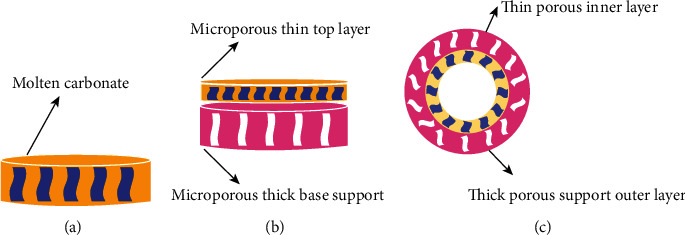
Different geometries of MC membranes. (a) Symmetric disc, (b) asymmetric disc, and (c) asymmetric tube.

Based upon the shape and geometry of support, membranes can be divided into three types. (a) symmetric disk, (b) asymmetric disk, and (c) asymmetric tubular membranes. Generally, the symmetric disk shape membranes are made up of one layer with molten-carbonate salt infiltrated homogenously in the membrane material (Figure 5(a)). They are prepared in the form of pellets using isotactic compression and tape casting methods at lab scale [25, 47].

These approaches are used because of ease in the preparation and experimentation at lab scale. However, CO_2_ permeation is limited in these symmetric disk-shaped membranes due to their significant thickness. Hence, advanced microstructural modifications have been applied for promoting thinner membranes such as asymmetric disks and hollow fibre/tubular membranes. Asymmetric disk type membrane consists of a thin dense membrane layer, infiltrated with molten salt, on a strong mechanical macroporous support (Figure 5(b)). Meanwhile, in asymmetric tubular geometry, thin, porous membrane layer exists as the inner layer of the tube and porous support forms outer layer of the tube (Figure 5(c)). For asymmetric geometry in oxygen ionic conducting membranes, reduction in membrane thickness enhances CO_2_ and O_2_ permeation flux [24, 48]. Furthermore, tubular structure offers higher surface area, easy scale-up, and convenient sealing procedure at high temperature [49].

**Figure 6:**
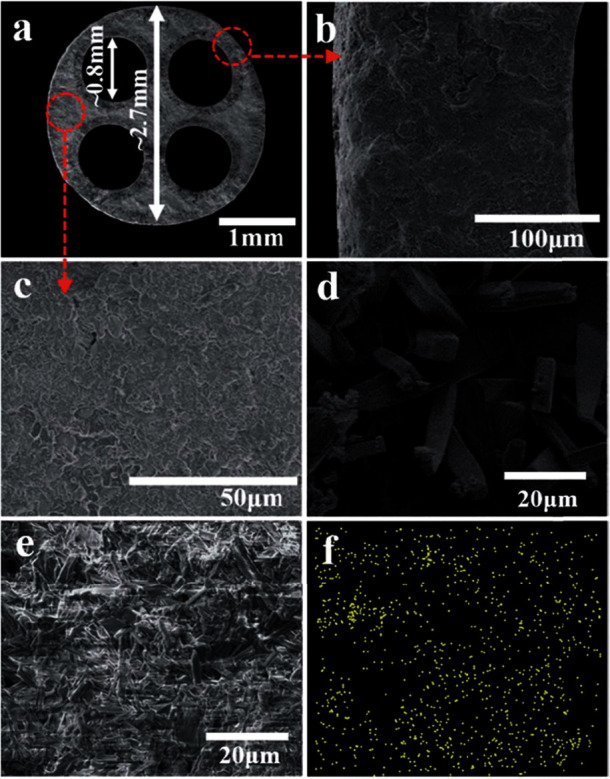
SEM images of SrFe_0.8_Nb_0.2_O_3-*δ*_ multihollow fibres with supported molten salt. (a) Cross-sectional view; (b) outer layer; (c) high-resolution cross-sectional view; (d) inner layer; (e) outer surface; (f) EDX of potassium in cross-sectional view [45].

Asymmetric tubular membranes are usually prepared by phase inversion, tape casting, spin spraying, and centrifugal casting techniques [48–51]. Recent literature reveals extensively the use of ceramic hollow fibres for molten-salt membrane support because of high surface area and reduced thickness of membrane. For YSZ and LSCF hollow fibre membranes, acceptable carbon dioxide fluxes have been reported [52, 53]. Fabrication of hollow fibre membrane is more manageable than pellet membranes, but single hollow fibre membrane possesses low mechanical strength and thermal stability. To solve this issue, multichannel hollow fibre membrane has been proposed, which consists of SrFe_0.8_Nb_0.2_O_3-*δ*_ and carbonates (Figure 6) with improved mechanical strength and high CO_2_ flux [45]. However, further research is required for determining new approaches to develop membrane supports for enhanced CO_2_ permeation with reduced thickness.

**Figure 7:**
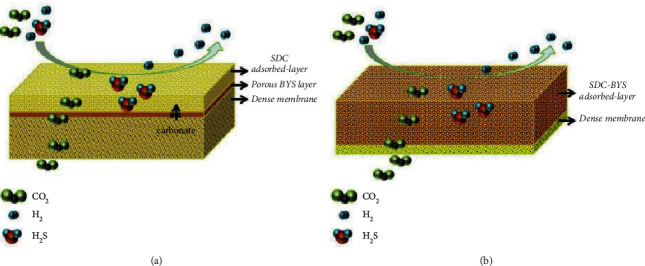
Asymmetric membranes for carbon capture in H_2_S gas environment: (a) three-layered; (b) two-layered [2].

Asymmetric membranes have been further modified by addition of a third layer to achieve stability under different operating environments such as H_2_S containing atmosphere. Chen et al. reported SDC-BYS-based three-layered asymmetric carbonate membrane, which showed excellent resistance against H_2_S gas [2]. In three-layered asymmetric membrane, two layers of SDC and BYS adsorb the H_2_S gas and prevent the third SDC dense carbonate layer from H_2_S attack (Figure 7(a)). Operational hours of three-layered SDC-BYS-SDC asymmetric membranes increased 10–12 times compared to single-layer SDC-BYS. The adsorbed layer can be regenerated in reduced atmosphere and trapped sulfur in this layer can be stripped out by converting into H_2_SO_4_, leading to reducing the cost of overall separation process. However, two-layered asymmetric membrane (Figure 7(b)) showed higher CO_2_ flux as compared to three-layered membrane which could be attributed to reduced thickness of dense layer.

#### 3.3.2. Porosity

**Figure 8:**
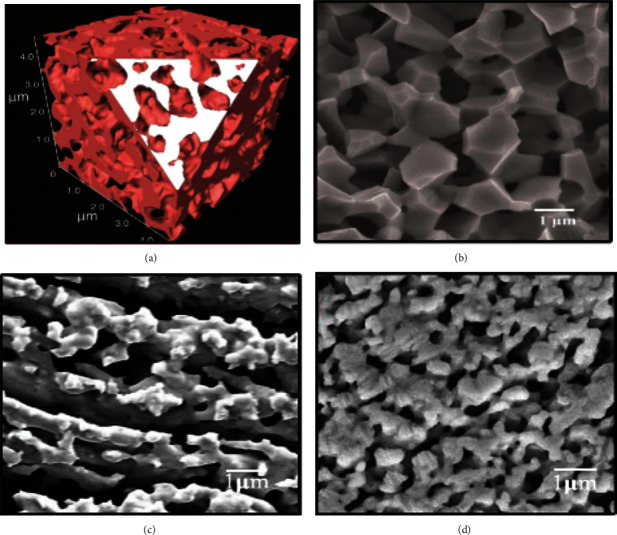
Reconstructed porous microstructure of SDC membrane: (a) 3D; (b) 2D SEM [26]; (c) porous Ag matrix after chemical dealloying [23]; (d) porous Ag matrix after electrochemical dealloying [28].

Carbon dioxide permeation is influenced by pore volume, pore size, pore connectivity, and tortuosity. These properties have great impact on support and molten-carbonate conductivity [54]. Overall, a thin support with highly interconnected uniformly distributed pores and low tortuosity is desirable. Gas-liquid interfacial area, triple-phase boundary length at feed and permeate side is also dependent upon surface porosity of the support. For pore formation, sacrificial materials such as metal oxides are used and porosity of support is controlled by varying quantity of sacrificial materials. After the formation of porous structure, sacrificial phase is removed by acid etching, firing (organic removal), or reduction [26, 55–57]. Zhang et al. used NiO as the sacrificial template in combination with coprecipitation of SDC precursors to synthesize a porous matrix support with compositional homogeneity and uniformly distributed pores for MOCC membrane [26].  Figure 8 portrays a sponge-like microstructure of SDC membrane, exhibiting high interconnectivity between pores and SDC matrix in 3D (a) and 2D (b) demonstration.

To create pores in metal supports such as Ag, for MECC type membranes, chemical-dealloying strategy has been applied. Fang et al. used alloy of Ag and Al, each with 50% composition with Al as the fugitive element [23]. After dealuminizing the samples in 3 M HCl solution for 48 hours, porous microstructures of Ag matrix were obtained, exhibiting pore size from 1 to 10 *μ*m (Figure 8(c)). Electrochemical dealloying using Zn as the sacrificial element has been employed further for producing single and more homogenous nanoporous Ag matrix (Figure 8(d)) [28]. This method is useful for formation of well-connected micron porous structure with uniform size distribution. Ability of molten salt retention is enhanced by formation of these submicron pores leading to increase in carbon dioxide flux [28, 57].

## 4. Performance Evaluation of MC Membranes

Different components of MC membranes are interrelated to one another in determining overall membrane performance in terms of CO_2_ flux measurement and stability at high temperature. This section presents overview of membranes in operation. Since there are various intrinsic parameters that influence membrane performance significantly and have a direct impact on CO_2_ permeation flux, membrane performance has been critically analysed based upon these parameters such as material selection, geometry, membrane thickness, support properties, and operating conditions (feed/sweep gas composition).

### 4.1. Effect of Material Selection and Geometry

#### 4.1.1. Mixed Electron and Carbonate Ion Conducting Support Membranes (MECC)

**Figure 9:**
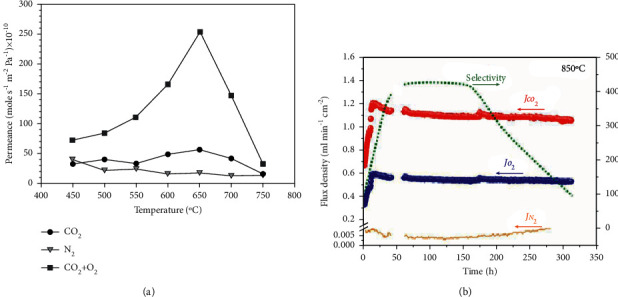
(a) Gas permeation of CO_2_, N_2_, and CO_2_ + O_2_ from a porous stainless steel support at different temperatures [12]. (b) Flux densities and selectivity of CO_2_ and O_2_ at 850°C with NiO support [35].

The concept of dual-phase membranes was first introduced by Chung et al. A ternary mixture of Li/Na/K carbonate was infiltrated in stainless steel support [12]. Rates of single gas permeation for CO_2_ and N_2_ were found to be very low for stainless steel support; however, the permeance of CO_2_ : O_2_ (2 : 1) was 0.13 ml·min^−1^·cm^−2^ at 650°C (Figure 9(a)).

Separation factor for CO_2_ over N_2_ with separately measured N_2_ permeation was found to be 16. However, the fluxes were found to be lower than expected because of interfacial reactions due to the formation of LiFeO_2_. Huang et al. applied Ag porous matrix with molten carbonate which increased CO_2_ flux 6 times, that is, 0.82 ml·min^−1^·cm^−2^ at 650°C as compared to stainless steel support membrane [27]. Ag exhibits high electron conductivity and better wettability with molten carbonates. However, CO_2_ flux decreases at higher temperatures due to sintering of Ag. Zhang and coworkers used NiO matrix as a support [35]. NiO showed enhanced CO_2_ flux up to 1.0 ml·min^−1^·cm^−2^ at 850°C and long-term stability at high temperature. This membrane showed good results for initial 15 hours of operation for both CO_2_ and O_2_ in the ratio of 2 : 1 (Figure 9(b)). After 15hours, permeation was slow because of interfacial reactions between the support and molten carbonate due to the formation of lithiated NiO layer of 100 nm thickness. CO_2_ fluxes for different types of MECC membranes are summarized in Table 2.

#### 4.1.2. Mixed Oxygen and Carbonate Ion Conducting Support Membranes (MOCC)

In MOCC membranes, CO_2_ is ionized only in the presence of O^2−^ ions and is converted to CO_3_^2−^ ions, consequently transported through the membrane. Thus, CO_2_ flux depends upon conductivity of O^2−^ ions. Various types of O^2−^ ion ceramic conductors have been applied in fabrication of MOCC membranes such as yttria-stabilized zirconia (YSZ) [47, 59], fluorite-structured Bi_1.5_Y_0.3_Sm_0.2_O_3_ (BYS) [42], samarium-doped ceria (SDC) [26, 60, 61], and gadolinium-doped ceria (GDC) [47]. Wade and coworkers compared CO_2_ flux measurements of molten carbonate membranes for YSZ and Al_2_O_3_. Al_2_O_3_-based membrane showed CO_2_ flux of 0.019 mL·min^−1^·cm^−2^, whereas YSZ-based membrane showed CO_2_ flux up to 0.13 mL·min^−1^·cm^−2^. The low flux in Al_2_O_3_-based membrane is because of nonoxide ion conducting nature of Al_2_O_3_ [47]. However, YSZ material itself has low O^2−^ ion conductivity as it reacts with LiCO_3_, leading to formation of lithium zirconate irreversibly at low pressure of CO_2_. Rui et al. applied a fluorite-structured BYS material to fabricate MC membrane, which showed CO_2_ flux of 0.083 mL·min^−1^·cm^−2^ [42].

SDC membranes doped with CeO_2_ have been investigated extensively because of their good stability, high O^2−^ conductivity, and better wettability with molten carbonate. However, there are various parameters that can affect membrane performance such as method of fabrication, geometry of support, thickness of support, and composition of feed gas. Zhang et al. developed SDC membrane of 200 *μ*m thickness using sacrificial template method [26]. This SDC membrane gave better CO_2_ flux (1.84 mL·min^−1^·cm^−2^) as compared to SDC membrane giving CO_2_ flux of 0.79 mL·min^−1^·cm^−2^ prepared by press-sintering method [60]. Enhanced CO_2_ flux could be attributed to the formation of intra- and interconnected ionic channels in membrane microstructure. CO_2_ fluxes for different types of MOCC membranes are reviewed in Table 3.

The thickness of ceramic supports has a significant impact on membrane performance. It has been observed that, in MOCC membranes, O^2−^ ion transport is the controlling factor; therefore, reduction in membraned thickness should enhance the ion transport, increasing CO_2_ flux consequently. Thin asymmetric membrane of 150 *μ*m thickness consisting of hermetic SDC− carbonate layer on the macroporous SDC/BYS base support showed higher CO_2_ flux (i.e., 0.88 mL·min^−1^·cm^−2^) as compared to CO_2_ flux of 0.79 mL·min^−1^·cm^−2^ for thick symmetric SDC geometry of 1500 *μ*m [60, 61].

**Figure 10:**
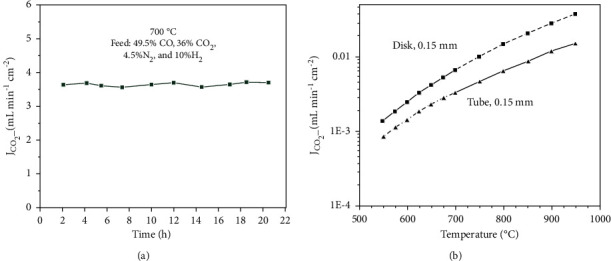
(a) High temperature stability test at 700°C for 0.12 mm asymmetric tubular dual-phase membrane in simulated syngas [62]; (b) comparison of CO_2_ fluxes for disk- and tube-shaped membranes as a function of temperature [61].

In addition to thickness, different shapes of porous supports have also been fabricated and tested to enhance CO_2_ permeation efficiency such as classical disc-shaped and tube-shaped MC membranes. In the past decade, most of the studies were focused on disc shape membranes. However, disc-shaped membranes offer limited area, whereas tubular/hollow fibre configuration is expected to enhance the flux because of high surface area and easy method of fabrication at high temperature [49]. Lin et al. fabricated thin asymmetric tubular membranes of 150 *μ*m and 120 *μ*m thickness, which showed significant increase in CO_2_ flux, that is, 1.56 mL·min^−1^·cm^−2^ and 2.05 mL·min^−1^·cm^−2^, respectively [49, 62]. The latter membrane configuration was tested at 700°C in 10% H_2_ in the feed gas in addition to N_2_, CO, and CO_2_ (Figure 10(a)). Among MOCC membranes, the highest CO_2_ flux of 5.46 mL·min^−1^·cm^−2^ has been reported by Chen et al. for a hollow fibre SDC− membrane; however, its stability was only up to 85 hours, which can be attributed to its low mechanical strength [63]. However, in few studies, disc-shaped membrane showed higher CO_2_ flux as compared to tube-shaped membrane despite of exhibiting same thickness [61]. The authors attributed the better performance to high particle packing density in disc samples as compared to the tubular membrane samples (Figure 10(b)).

#### 4.1.3. Mixed Electron, Oxygen, and Carbonate Ion Conducting Membranes (MEOCC)

**Figure 11:**
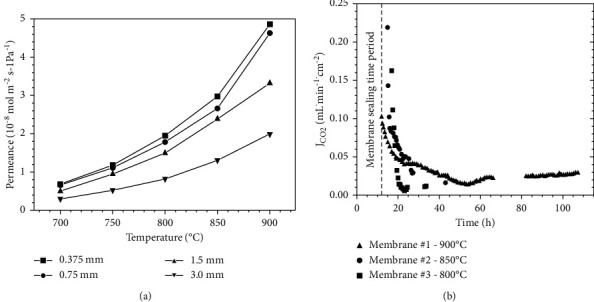
(a) Effect of membrane thickness as a function of temperature on CO_2_ permeance [24]; (b) change in CO_2_ permeance as a function of time at different temperatures in the absence of O_2_ [64].

In MEOCC membranes, CO_3_^2−^ ions are formed by two types of reactions: (a) CO_2_ + O^2−^ ⟶ CO_3_^2−^ and (b) CO_2_ + 1/2O_2_ + 2e^−^ ⟶ CO_3_^2−^. Therefore, ceramic support with conduction of both O^2−^ ions and electrons is required. Perovskites-structured oxides possess high O^2−^ ions and electron conduction. Various types of perovskite oxides have been experimented to fabricate MEOCC membranes such as La_0.6_ Sr_0.4_ Co_0.8_ Fe_0.2_ O_3-*δ*_ (LSCF) [24, 64], La_0.5_ Sr_0.5_ Fe_0.8_ Cu_0.2_ O_3-*δ*_ (LSFCu) [65], SrFe_0.8_ Nb_0.2_ O_3-*δ*_ (SFN) [45], and La_0.85_ Ce_0.1_ Ga_0.3_ Fe_0.65_ Al_0.05_ O_3-*δ*_ (LCGFA) [25]. Anderson and Lin applied LSCF material and obtained a CO_2_ flux of 0.3 mL·min^−1^·cm^−2^ (4.77 × 10^−8^ mol·m^−2^·s^−1^·Pa^−1^) at 900°C for 375 *μ*m thick support membrane [24]. Moreover, the effect of membrane thickness on CO_2_ permeance was also investigated systematically for MEOCC membranes. It had been observed that reducing membranes thickness enhanced CO_2_ permeance as surface reaction becomes significant on decreasing thickness (Figure 11(a)). However, LSCF was found to be unstable in an O_2_-free environment [64].  Figure 11(b) shows a fast decay in CO_2_ flux, showing a steady-state value of 0.03 mL·min^−1^·cm^−2^ after 65 hours' exposure at 900°C. This could be attributed to the layer of SrCO_3_ formed on the membrane surface by reaction of LSCF with CO_2_. This can be prevented by adding O_2_ in the feed gas along with CO_2_. In the presence of O_2_, SrCO_3_ is decomposed to SrO at 800°C, which would protect the LSCF material beneath [66]. CO_2_ fluxes for different types of MEOCC membranes are summarized in Table 4.

Norton et al. achieved CO_2_ flux up to 3.0 mL·min^−1^·cm^−2^ by introducing O_2_ in the feed gas in addition to CO_2_ and N_2_. The presence of O_2_ improved the membrane stability, maintaining the ionic and electronic conductivity of LSCF as well.

In [64], Lin and coworkers applied La_0.5_Sr_0.5_Fe_0.8_Cu_0.2_O_3-*δ*_ (LSFCu) to develop MEOCC membrane. Maximum flux of 1.55 ml·min^−1^·cm^−2^ was achieved at 650°C in 20% CO_2_/80% O_2_ mixture of feed gas. The mechanism of CO_2_ permeation was attributed to coherent interaction of CO_3_^2−^ and O^2−^ ions [65]. Perovskite material with A-site fee alkaline is known to be stable against reaction with CO_2_ at high temperature. In this regard, La_0.85_ Ce_0.1_ Ga_0.3_ Fe_0.65_ Al_0.05_ O_3-*δ*_ (LCGFA) was found to be compatible with molten-carbonate salts and chemically stable at high temperature, that is, 900°C [25]. However, the CO_2_ flux (0.044 ml·min^−1^·cm^−2^) obtained for LCGFA was much lower than that of LSCF membranes.

**Figure 12:**
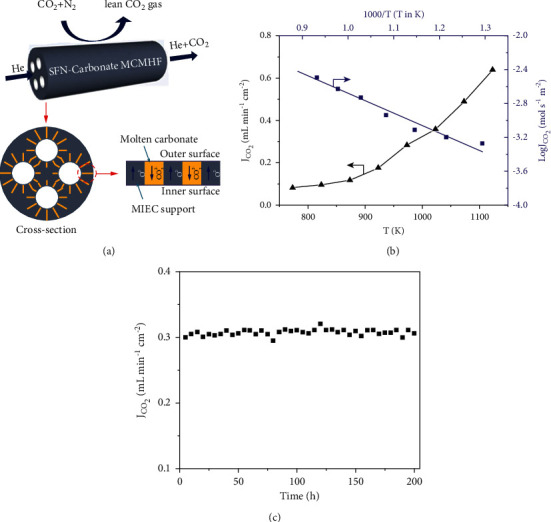
(a) Structure of multichannel hollow fibre SNF membrane. (b) Effect of temperature on CO_2_ permeation flux of SFN-MC membrane. (c) Stability test: CO_2_ permeation flux as a function of time [45].

Support geometry other than disk shape has also been applied for MEOCC membranes such as multichannel hollow fibres. Asymmetric hollow fibre-based membranes (Figure 12(a)) offer high surface/volume ratio, less transport resistance, and easy method of sealing. CO_2_ permeation flux of 0.64 mL·min^−1^·cm^−2^ at 850°C was reported by Jiang et al. for multichannel hollow fibre membrane of SrFe_0.8_ Nb_0.2_ O_3-*δ*_ (SFN) support material with 220 *μ*m (Figure 12(b)) [45]. Meanwhile stability test showed that CO_2_ permeation flux was maintained at of 0.31 mL·min^−1^·cm^−2^ for 200 hr at 700°C (Figure 12(c)). SFN membrane showed good chemical stability as well.

Combination of fluorite and perovskite conducting material has also been used as ceramic support for MEOCC membranes such as Ce_0.85_Sm_0.15_O_2-_Sm_0.6_Sr_0.4_Al_0.3_Fe_0.7_O_3_ (SDC-SSAF) composite material [46]. These composite dual-phase membrane matrices are known to exhibit high CO_2_ permeability and significant chemical resistance against CO_2_ attack [67]. SDC-SSAF composite membrane of thickness of 1300 *μ*m showed CO_2_ flux of 0.24 mL·min^−1^·cm^−2^ at 900°C, which increased up to 0.28 mL·min^−1^·cm^−2^, when O_2_ was added in the feed gas [46].

### 4.2. Effect of Membrane Thickness

CO_2_ flux for any membrane is controlled by surface reaction and bulk diffusion [68]. Effect of membrane thickness on CO_2_ flux is summarized in Table 5. From equation (3), it can be seen that permeation flux is inversely proportional to thickness. The general trend observed in MC membranes is that decreasing membrane thickness would enhance surface reactions, increasing CO_2_ flux consequently. There is a critical thickness, below which surface exchange reaction becomes slow. A critical thickness of 840 *μ*m is reported for Ag-carbonate MECC membrane [58]. Above 840 *μ*m, decrease in thickness increases CO_2_ flux. However, decrease in membrane thickness does not cause the proportionate rise in CO_2_ flux as expected, in case of MOCC membranes. Lin et al. reported CO_2_ flux of 0.17 ml·min^−1^·cm^−2^ and 0.87 ml·min^−1^·cm^−2^ for thick symmetric SDC membrane (1500 *μ*m) and thin asymmetric SDC membrane (150 *μ*m), respectively [60, 61]. The increase in CO_2_ flux was less than expected, that is, only 5 times as compared to 10 times increase in thickness, suggesting the limitation from surface exchange reaction. Inverse relation between membrane thickness and flux is further evident from activation energy values. Activation energies of thin asymmetric membranes (150 *μ*m: 60.3 kJ·mol^−1^ and 120 *μ*m: 62.5 kJ·mole^−1^) are lower than that of thick symmetric membranes (1500 *μ*m: 81.2 kJ·mol^−1^ and 1000 *μ*m: 82.4 kJ·mol^−1^) [49, 62]. Difference in activation energies could be attributed to the difference in porosity and tortuosity of the membrane materials, attained during fabrication procedures such as sintering conditions, which ultimately affects conduction of oxygen and carbonate ions [54].

In case of MEOCC membranes such as LSCF-MC membrane, the trend of activation energy change is opposite. Activation energies tend to increase with decreasing membrane thickness, while CO_2_ flux increases with decreasing thickness (Table 4). Increase in activation energies can be attributed to the involvement of surface exchange reactions on decreasing thickness [24]. However, for LCGFA-MC membrane, activation energy values have been found to be constant for both 750 and 1500 *μ*m thick membranes, whereas CO_2_ flux is higher for thinner membrane, suggesting that CO_2_ flux is controlled by bulk diffusion rather than surface exchange reaction [25].

### 4.3. Effect of Support Microstructure

The microstructure properties of supports such as porosity, pore size distribution, tortuosity, and density of triple-phase boundary play a significant role in evaluating membrane performance because the porous support in molten-carbonate membranes not only supports MC phase but also provides medium for conduction of O^2−^ ions and electrons. Ortiz-Landeros et al. investigated that by decreasing the sintering temperature of LSCF-MC membrane from 1100 to 1000°C.

CO_2_ flux was increased three times [54]. This can be attributed to the changes in microstructure at low sintering temperature such as increase in porosity and decrease in tortuosity. Porosity can also be enhanced in matrix supports using pore forming agents such as carbon, cellulose, and metal oxides. Zhang et al. prepared a series of interconnected three-dimensional SDC supports of different porosity using NiO as a sacrificial pore former agent. With increase in porosity from 30 to 50%, tortuosity of the membrane decreased from 26.1 to 2.2 and CO_2_ flux increased from 0.26 to 1.84 ml·min^−1^·cm^−2^ [26].

CO_2_ flux and long-term stability of MC membranes is also influenced by pore size of the matrix. Capillary forces cause retention of carbonate phase in porous matrix, which depend upon pore size consequently. Large pores cannot generate capillary forces to withhold MC phase, leading to loss of MC phase and reduction in CO_2_ flux. Large pores also reduce the density of triple-phase boundaries, causing decrease in CO_2_ flux. Different types of pore formers and fabrication methods have been employed to reduce pore size for MC membranes (Table 6). By changing the pore former from cellulose (pore size: 15–20 *μ*m) to carbon (pore size: 8–10 *μ*m), CO_2_ flux was increased from 0.39 to 0.61 ml·min^−1^·cm^−2^ in Ag-MECC membranes [58, 69]. Fang and coworkers further decreased the pore size to 1 *μ*m using electrochemical dealloying process for pore formation and achieved CO_2_ flux up to 1.02 ml·min^−1^·cm^−2^ [23]. Same trend was observed by Zhang et al. for SDC membrane, where pore size was reduced to 0.55 *μ*m along with subsequent increase in porosity leading to high CO_2_ flux [26]. It can also be inferred from Table 6 that long term stability of MC membrane is enhanced by decreasing pore size of matrix support. This is because small pore size retains MC phase much longer at high temperatures, increasing CO_2_ flux ultimately.

### 4.4. Effect of Feed/Sweep Gas

Compositions of feed and sweep gas influence CO_2_ transport across MC membranes. Various research efforts have been dedicated to studying the effects of different gases used as feed/sweep on CO_2_ flux (Table 7). It can be inferred from equation (3) that partial pressure difference of CO_2_ across the membrane at feed and permeate side has a significant impact on its transport in MC membranes. The higher the partial pressure gradient of CO_2_ was, the higher would the CO_2_ flux be.

**Figure 13:**
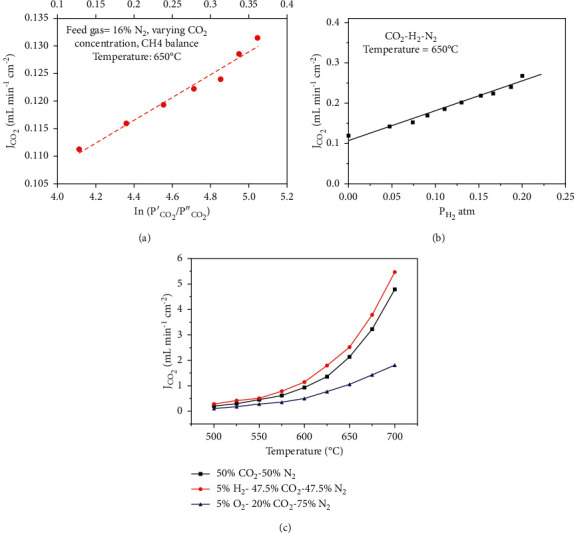
CO_2_ flux density of SDC membrane (a) as a function of logarithm of CO_2_ partial pressure [70], (b) as a function of H_2_ partial pressure [26], and (c) in the presence and absence of H_2_ [63].

Norton et al. studied the effect of increasing CO_2_ partial pressure on SDC− membrane and found that, by increasing the CO_2_ pressure at feed side from 0.1 to 0.9 atm, CO_2_ flux across the membrane also increased from 0.39 to 0.79 ml·min^−1^·cm^−2^ [60]. MOCC membranes have been assessed in CH_4_ gas environment. SDC-MC membrane of 1150 *μ*m thickness has been tested to capture CO_2_ using CH_4_ gas in the feed. CO_2_ flux was measured at different partial pressures of CO_2_ at feed side. Highest CO_2_ flux of 0.13 ml·min^−1^·cm^−2^ was obtained at CO_2_ partial pressure of 0.375 atm (Figure 13(a)) [70]. Thus, MOCC membranes can also be employed in purification of shale gas/biogas.

CO_2_ flux of MC membranes can also be enhanced by decreasing partial pressure of O_2_ at feed or sweep side such as adding H_2_ on feed side. Since O^2−^ ions travel from sweep to feed side in membrane, addition of H_2_ would decrease partial pressure at feed side causing rise in O^2−^ ion flux, thus leading to corresponding increase in CO_2_ flux in opposite direction [16]. Since MOCC membranes involve transport of O^2−^ ions, they are more appropriate to capture CO_2_ in feed environments containing H_2_ such as from precombustion processes.  Figure 13(b) shows linear relationship between CO_2_ flux and partial pressure of H_2_ at feed side for SDC membrane [26]. Chen and coworkers measured CO_2_ flux of 4.78 ml·min^−1^·cm^−2^ and 5.46 ml·min^−1^·cm^−2^ in the absence and presence of 5% H_2_ in feed gas, respectively, for an SDC hollow fibre membrane of 100 *μ*m thickness (Figure 13(c)) [63].

For MECC membranes, CO_2_ flux can be enhanced by adding H_2_ to sweep side. Here the mechanism is different from that of MOCC membranes. H_2_ reacts with the permeated O_2_, causing decrease in O_2_ concentration. This would shift the equilibrium to the right side (CO_3_^2−^⇌CO_2_+1/2O_2_+2e^−^), followed by increase in CO_2_ permeation. Fang et al. investigated that, by increasing H_2_ to 1.41% in sweep gas mixture, CO_2_ flux could be enhanced to 2 times (1.02 ml·min^−1^·cm^−2^) as compared to CO_2_ flux in pure Ar (0.49 ml·min^−1^·cm^−2^) for Ag-MC membrane [23].

### 4.5. Effect of Surface Modification

Surface modification of membranes can be employed to facilitate surface reaction without changing the bulk properties. Various methods have been applied to modify surface such as colloidal deposition, chemical vapor deposition, and atomic layer deposition. This section elaborates in detail on each method.

#### 4.5.1. Colloidal Deposition

**Figure 14:**
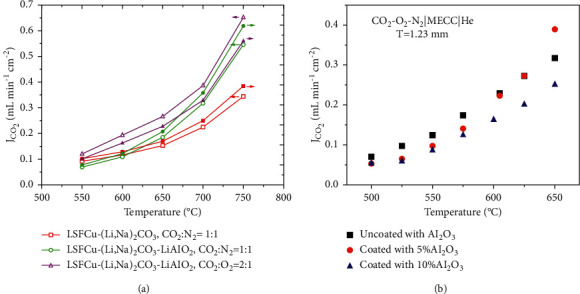
(a) CO_2_ fluxes for LSFCu-(Li, Na)_2_CO_3_ and LSFCu-(Li, Na)_2_CO_3_ samples modified with LiAlO_2_ as a function of temperature [65]. (b) CO_2_ fluxes for Ag-MC membranes coated with Al_2_O_3_ as a function of temperature [69].

LiAlO_2_ is a material of choice for surface modification as it has good wetting compatibility with molten carbonate and better surface adsorption for CO_2_. Lan et al. introduced 10 wt% LiALO_2_ in La_0.5_Sr_0.5_Fe_0.8_Cu_0.2_O_3-*δ*_ (LSFCu)-(Li, Na)_2_CO_3_ MEOCC composite membrane. Deposition of LiALO_2_ increased the CO_2_ permeation flux from 0.35 to 0.55 ml·min^−1^·cm^−2^ (Figure 14(a)) at 750°C because of enhancement of surface reactions [65]. Surface of Ag-MC membrane was also coated with a thin layer of colloidal *γ*-Al_2_O_3_ solution before impregnation with molten-carbonate salt [69]. MC salt is expected to react with Al_2_O_3_ to form a layer of LiAlO_2_ on the surface, enabling better wettability between molten carbonate and Ag porous network. However, the Ag-MC membrane coated with 5% Al_2_O_3_ gave the highest CO_2_ flux (0.39 ml·min^−1^·cm^−2^) and stability as compared to that coated with 10% Al_2_O_3_ and uncoated sample (Figure 14(b)). 5% Al_2_O_3_ was the optimum concentration limit, above which bulk transport CO_3_^2−^ may be hindered, leading to reducing the CO_2_ flux.

#### 4.5.2. Chemical Vapor Deposition (CVD)

**Figure 15:**
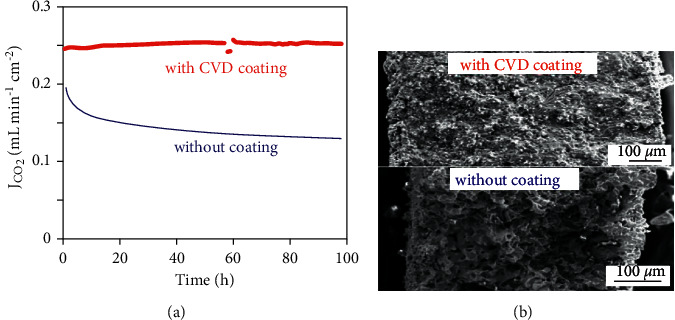
(a) CO_2_ flux versus time relation for samples coated with and without CVD at 650°C. (b) SEM images for samples coated with and without CVD [34].

Chemical vapor deposition (CVD) involves coating of thin films on a heated substrate by means of gaseous phase precursors and coating can be obtained with tunable deposition rates [71]. In Al_2_O_3_ modified Ag-MC membranes, with colloidal deposition of Al_2_O_3_, usually the thickness of coating cannot be controlled and subsequently stability improvements cannot be consistent from batch to batch. A uniform layer of Al_2_O_3_ was deposited over the surface of Ag matrix by means of CVD, which enhanced the stability of MECC membrane [34]. No sign of degradation was observed for 100 hours at 650°C as compared to the pristine sample which lost 50% of its original flux in the first 20 hours (Figure 15(a)). Large pores in SEM images further indicate significant sintering of Ag particles and loss of metal carbonate in pristine sample, while the presence of dense microstructures in CVD coated sample shows decrease in sintering (Figure 15(b)).

#### 4.5.3. Atomic Layer Deposition (ALD)

Atomic layer deposition (ALD) is a technique used for ultrathin films formation by gas phase deposition with thickness control at submicron and nanometer levels at wide temperature range [20]. ALD process involves use of precursors segregated from each other in a gas phase by purging and pulsing alternatively [72]. Due to consecutive pulsing, monolayer can be formed in each ALD cycle, and its thickness can be tuned by replicating ALD cycles. ALD is highly favourable for porous materials because of the following reasons:

- Precursors used in ALD can be tuned into very small pores because of their vaporization phase. These small pores can be adsorbed on pore walls and rejoin with previously formed precursors [73].
- ALD occurs on surface of substrate, so very-high-quality and uniform thin film is deposited on highly dense ceramic porous support [74].
- By changing the ALD cycles, thickness of the layers can be accurately controlled [75].

**Figure 16:**
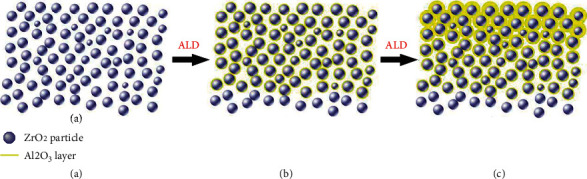
Schematics of pore tailoring by ALD of alumina over zirconia: (a) pristine membrane before ALD operation; (b) application of ALD; (c) increasing alumina thickness with increasing ALD cycles [76].

ALD technique has been applied to fabricate ceramic membranes. Li et al. reported application of ALD for ceramic membranes which consisted of zirconia nanoparticles sintered on alumina supports. Precise pore tailoring was achieved by modification of uniform and conformal layer of metal oxides on BSA (bovine cerium and radium) ceramic support [76].  Figure 16 clearly indicates that a number of ALD cycles increase the thickness of deposited layer of alumina which increases the grain size leading to decrease in pore size subsequently. For maximum efficiency, porosity of membrane can be tuned by selecting specific ALD cycle.

**Figure 17:**
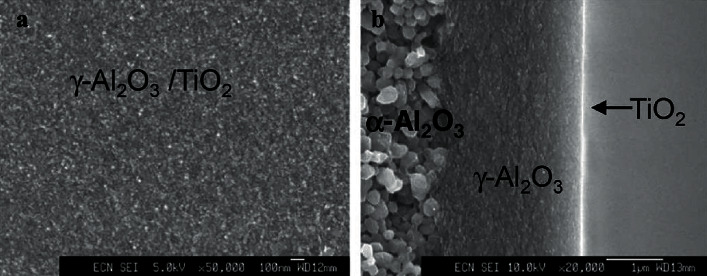
SEM images of *γ*-Al_2_O_3_/TiO_2_ membrane using PE-ALD: (a) top surface and (b) cross section [77].

Tran et al. fabricated alumina-titania composite membrane for H_2_ separation by means of plasma-enhanced ALD (PE-ALD). A thin titania layer (approximately 10 nm thickness) was deposited on *γ*-Al_2_O_3_ support by adjusting the number of ALD cycles (280 cycles) [77]. SEM image in Figure 17(a) shows the top view of *γ*-Al_2_O_3_ which appears smooth with no pinholes. Although the TiO_2_ layer deposited through PE-ALD is not clearly visible, the deposition of TiO_2_ seems to be uniform and shows no significant effect on *γ*-Al_2_O_3_ layer (Figure 17(b)). These membranes were tested under thermal and hydrothermal conditions for high CO_2_/H_2_ selectivity in steam reforming and water-gas shift processes for H_2_ gas separation [78].

**Figure 18:**
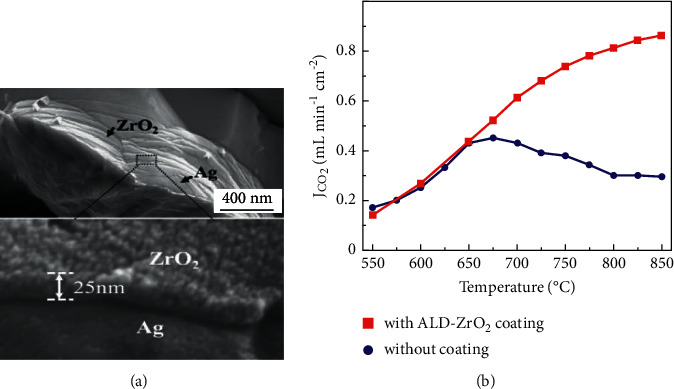
(a) SEM image of ALD deposited ZrO_2_/Ag membrane. (b) CO_2_ fluxes of ZrO_2_/Ag membrane with and without ALD-ZrO_2_ coating [36].

ALD treatment has also been applied in Ag-MC membranes for CO_2_ separation to further minimize the sintering problems of Ag. Zhang et al. deposited 25 nm thick ZrO_2_ layer on Ag matrix using 200 cycles of ALD [36]. The ZrO_2_ film was uniform and dense and adhered strongly to Ag matrix (Figure 18(a)). It not only increased CO_2_ flux up to 0.8 ml·min^−1^·cm^−2^ but also led to much stabilized Ag matrix resistant to sintering resulting in prolonged operational hours (850 hours) at 700°C (Figure 18(b)).

## 5. Major Challenges and Prospective Solutions

Molten-carbonate membranes offer significant potential to separate CO_2_ at high temperature, due to their inherent characteristics such as permeability, selectivity, reproducibility, and high temperature stability. However, their potential to be cost-effective and energy-efficient is still not investigated. In addition, their application at commercial scale is also limited till now, as most of the research work has been dedicated to their fabrication and testing at lab scale. This section highlights the key areas of deficiencies of molten-carbon membranes including long-term stability, their methods of fabrication, material selection, and commercialization.

### 5.1. Long-Term Stability at High Temperature

In current scenario of the research related to molten-carbonate membranes, the most crucial challenge is to achieve and maintain maximum efficiency in terms of CO_2_ permeation flux for longer operational hours at high temperature. In this regard, long-term stability is more essential than achieving just high values of CO_2_ fluxes. Up till now, the longest stability achieved at lab scale testing is 1000 hours approximately. The main causes of membrane degradation are loss of molten-carbonate phase at high temperatures and sintering of microporous supports, leading to decrease in CO_2_ flux, which ultimately requires to direct research efforts at improving all components and mechanisms of MC membranes.

### 5.2. Material Selection

Molten-carbonate membranes consist of a microporous solid support infiltrated with molten-carbonate salts. Among MECC membranes, Ag has shown promising results in terms of increasing CO_2_ flux; however, sintering of Ag at high temperature and its high cost limits its application at industrial level. Although surface modifications have been applied to reduce degradation of Ag, high magnitudes of CO_2_ fluxes have still not been achieved as compared to those of MOCC membranes. Other materials such as NiO showed better fluxes in MECC membranes; however, poor stability of NiO in reducing atmospheres limits its use at industrial level. Therefore, finding new materials in MECC membranes is highly desirable.

For MOCC membranes, many materials have been investigated, which allow high conduction of O^2−^ ions, such as SDC and GDC. However, they degrade in flue gas environments containing SO_2_ and H_2_S impurities. Among MEOCC membranes, majority of the research work has been conducted on LSCF type material. Overall, CO_2_ fluxes achieved for MEOCC membranes are lower than those for MOCC membranes with no substantial improvement in long-term stabilities at high temperatures. Thus, new materials with good chemical stability and better shelf-life are needed to be explored for both MOCC and MEOCC membranes.

### 5.3. Microporous Support/Geometry/Surface Modifications

Numerous research papers have been dedicated to optimizing the microporous structure and geometry of support. The general agreement is that molten-carbonate membrane exhibiting reduced thickness, high porosity, low tortuosity, and well-connected uniformly distributed pores of small size would promote CO_2_ permeation. Overall, reduction in pore size and formation of well-connected three-dimensional porous structure has enhanced long-term stability of membranes, but reduction in membrane thickness is limited by surface exchange reactions. In addition, asymmetric geometries such as hollow fibres showed highest CO_2_ fluxes because of high surface area. However, they exhibit low mechanical strength, which limits their utilization. Tailoring molten-carbonate membranes through surface modifications such as CVD and ALD has not only improved CO_2_ flux but also increased stability in MECC membranes. Surface modification strategies should be applied to MOCC and MEOCC membranes as well to further tune support properties such as pore size and pore volume.

### 5.4. Fabrication Methods and Commercialization

In view of optimizing support structure and geometry of molten-carbonate membranes, many fabrication techniques have been used to improve CO_2_ flux and stability such as tape casting, extrusion, phase inversion, centrifugal casting, and sacrificial-template synthesis. However, at present, most of these fabrication methods are limited to lab scale. Their complexity, high cost, and viability at industrial scale are still not evaluated in systematic manner. More efforts are needed not only to develop cost-effective methods of fabrication but also to make them compatible to capture CO_2_ in industry according to the type of environment such as precombustion processes, water gas shift reactions (WGS), dry methane reforming (DMR), and biogas purification systems.

## 6. Conclusions

This review summarizes the recent progress in molten-carbonate membranes with focus on material selection, geometry, and surface modifications. Based upon mechanism, three types of membranes have been reviewed thoroughly, that is, MECC, MOCC, and MEOCC. In addition, the impact of physical properties of membranes (support microstructure, geometry, and membrane thickness) and operating conditions (feed/sweep gas composition and presence of impurities) on membrane performance has also been discussed in detail.

MC membranes have been found to be quite promising for CO_2_ permeation because their intrinsic properties such as selectivity, permeability, and scalability can be easily tailored and fine-tuned by optimizing fabrication methods with operating conditions. However, the key challenges such as long-term stability at high temperatures, feasibility at commercial scale, and cost-effectiveness need to be addressed systematically. Moreover, selection of alternative materials and integration of fabrication methods with surface modifications (e.g., ALD) are highly recommended to improve CO_2_ permeation along with chemical and long-term stability at lab scale.

## Figures and Tables

**Table 1:** Melting points of pure salts of carbonates and various eutectic mixtures [29].

Salt	Melting point
Li_2_CO_3_	723
Na_2_CO_3_	854
K_2_CO_3_	891
Li_2_CO_3_-Na_2_CO_3_ (52 : 48) mol %	501
Li_2_CO_3_-K_2_CO_3_ (62 : 38) mol %	498
Na_2_CO_3_-K_2_CO_3_ (56 : 44) mol %	710
Li_2_CO_3_-Na_2_CO_3_-K_2_CO_3_ (43.5 : 31.5 : 25) mol %	397

**Table 2:** Comparison of CO_2_ fluxes and stabilities for different types of MECC membranes.

Support material/geometry	Fabrication method	Thickness (*μ*m)	Feed gas/sweep gas	CO_2_ flux/temp. (ml·min^−1^·cm^−2^)/(°C)	Stability	Reference
Stainless steel/Li : Na : K ∗Sym. disc	Press-sintering	1570	CO_2_ : O_2_ = (2 : 1)/vacuum	0.13/650		[12]

Ag/Li : K Sym. disc	Press-sintering	1670	CO_2_ : O_2_ : N_2_ = (5 : 5 : 2)/He	0.82/650	80 hr at 750°C	[27]

Ag/Li : K (Ag coated with Al_2_O_3_) Sym. disc	Press-sintering	630	CO_2_ : O_2_ : N_2_ = (5 : 5 : 2)/He	0.61/600	326 hr at 600°C	[58]

Ag/Li : Na Sym. disc	Press-sintering/sacrificial chemical dealloying	960	CO_2_ : O_2_ : N_2_ = (3 : 2 : 15)/9.4% H_2_-Ar	1.02/600	900 hr at 600°C	[23]

Ag/Li : Na Sym. disc	Press-sintering/sacrificial electrochemical dealloying	910	CO_2_ : O_2_ : N_2_ = (3 : 2 : 15)/9.4% H_2_, Ar	0.89/650	500 hr at 600°C	[28]

NiO/Li : Na Sym. disc	Press-sintering	1200	CO_2_ : O_2_ : N_2_ = (3 : 2 : 15)/Ar	1.0/850	320 hr at 850°C	[35]

∗Sym.: symmetric.

**Table 3:** Comparison of CO_2_ fluxes and stabilities for different types of MOCC membranes.

Support material/geometry	Fabrication method	Thickness (*μ*m)	Feed gas/sweep gas	CO_2_ flux/temp. (ml min^−1^·cm^−2^)/(°C)	Stability	Reference
YSZ/Li : Na:K Sym. disc	Press-sintering		CO_2_ : N_2_ = (1 : 1)/He	0.01/650		[59]

YSZ/Li : Na : K Sym. disc	Press-sintering	250	CO_2_ : He = (1 : 1)/Ar	0.13/750	66 hr at 750°C	[47]

BYS/Li : Na : K Sym. disc	Press-sintering	50	CO_2_ : Ar = (1 : 1)/He	0.083/650	70 hr at 650°C	[42]

SDC/Li : Na : K Sym. disc	Coprecipitation and sacrificial template	1200	CO_2_ : H_2_ : N_2_ = (10 : 1 : 10)/He	1.84/700		[26]

SDC/Li : Na : K Sym. disc.	Press-sintering	1500	CO_2_ : CO : H_2_ : N_2_ = 7 : 10 : 2:1/He	0.79/900	840 hr at 700°C	[60]

SDC/SDC-BYS/Li : Na : K ∗Asym. tube	Centrifugal casting	150	CO_2_ : N_2_ = (1 : 1)/He	1.56/900		[49]

SDC/SDC-BYS/Li : Na : K Asym. disc	Press-sintering	150	CO_2_ : N_2_ = (1 : 1)/He	0.88/700	160 hr at 700°C	[61]

SDC/SDC-BYS/Li : Na : K Asym. tube	Centrifugal casting	120	CO_2_ : CO : H_2_ : N_2_ = 7 : 10 : 2:1/He	2.05/900	22 hr at 700°C Stable in syngas	[62]

SDC/Li : Na Hollow fibre	Phase inversion	100	CO_2_ : H_2_ : N_2_ = (10 : 1 : 10)/He	5.46/700	85 hr at 600°C	[63]

∗Asym.: asymmetric.

**Table 4:** Comparison of CO_2_ fluxes and stabilities for different types of MEOCC membranes.

Support material/geometry	Fabrication method	Thickness (*μ*m)	Feed gas/sweep gas	CO_2_ flux/temp. (ml·min^−1^·cm^−2^)/(°C)	Stability	Reference
LSCF/Li : Na : K Sym. disc	Press-sintering	375	CO_2_ : Ar = (1 : 1)/He	0.32/900		[24]

LSCF/Li : Na : K Sym. disc	Press-sintering	1000	CO_2_ : N_2_ = (1 : 1)/Ar CO_2_ : O_2_ : N_2_ = (2 : 1:1)/Ar	0.02/700 0.051/900	110 hr at 900°C 600 hr at 850°C	[64]

LSFCu/Li : Na Sym. disc	Press-sintering	1500	CO_2_ : N_2_ = (1 : 1)/He CO_2_ : O_2_ = (1 : 4)/He	0.15/650 1.55/750		[65]

LCGFA/Li : Na : K Sym. Disc	Press-sintering	750	CO_2_ : N_2_ = (1 : 1)/Ar	0.044/900	275 hr at 900°C	[25]

SFN/Li : Na : K Multichannel hollow tube	Phase inversion and sintering	220	CO_2_ : N_2_ = (1 : 1)/He	0.31/700	200 hr at 700°C	[45]

SDC-SSAF/Li : Na : K Sym. disc	Press-sintering	1300	CO_2_ : He : N_2_ = (3 : 3 : 14)/N_2_ CO_2_ : O_2_ : He = (15 : 6 : 15)/N_2_	0.24/900 0.28/900		[46]

**Table 5:** Comparison of CO_2_ fluxes for MC membranes with different thickness.

Support material	Symmetry	Thickness (*μ*m)	CO_2_ flux/temp. (ml min^−1^·cm^−2^)	Activation energy (kJmol^−1^)	Reference
Ag/Li : K (Ag coated with Al_2_O_3_) Sym. disc (MECC)	Sym. disc	630 840 1140 1210 1450	0.61 0.61 0.32 0.28 0.23		[58]

SDC/SDC-BYS/Li : Na : K (MOCC)	Asym. disc Sym. disc	150 1500	0.87 0.17	63	[61] [60]

SDC/SDC-BYS/Li : Na : K (MOCC)	Asym. tube Sym. tube	150 1500	1.56 0.51	60.3 81.2	[49] [49]

SDC/SDC-BYS/Li : Na : K (MOCC)	Asym. tube Sym. tube Sym. tube	120 1000 1500	2.05 0.6 0.5	62.5 82.4 80.4	[62]

LSCF/Li : Na : K (MEOCC)	Sym. disc	375 750 1500 3000	0.32 0.31 0.25 0.14	89.9 89.6 87.7 86.4	[24]

LCGFA/Li : Na : K (MEOCC)	Sym. disc	750 1500	0.044 0.024	96 96	[25]

**Table 6:** Comparison of CO_2_ fluxes for MC membranes with support microstructures of different pore sizes.

Support material/geometry	Fabrication method	Pore size (*μ*m)	Thickness (*μ*m)	CO_2_ flux (ml·min^−1^·cm^−2^)	Stability	Reference
Ag/Li : K (Ag coated with Al_2_O_3_) Sym. disc	Sacrificial press-sintering (pore former: cellulose)	15–20	1230	0.39	130 hr at 600°C	[69]

Ag/Li : K (Ag coated with Al_2_O_3_) Sym. disc	Sacrificial press-sintering (pore former: carbon)	8–10	630	0.61	326 hr at 600°C	[58]

Ag/Li : Na Sym. disc	Sacrificial press-sintering/chemical dealloying	1	960	1.02	900 hr at 600°C	[23]

SDC/Li : Na : K Sym. disc	Coprecipitation and sacrificial template: pore former: NiO	0.55	1200	1.84		[26]

**Table 7:** Effect of feed/sweep gas on CO_2_ fluxes of MC membranes.

Support material/geometry	Thickness (*μ*m)	Feed gas	Sweep gas	CO_2_ flux/temperature (ml·min^−1^·cm^−2^)/°C	Reference
SDC/Li : Na : K Sym. disc.	1500	CO_2_ : CO : H_2_ : N_2_ = 7 : 10 : 2:1	He	0.79 (*P*_CO2_ : 0.9 atm)/900 0.39 (*P*_CO2_ : 0.1 atm)/900	[60]

SDC/Li : Na : K Sym. disc.	1150	CO_2_ : CH_4_ : N_2_ = 3 : 14 : 2	Ar	0.13 (*P*_CO2_ : 0.375 atm)/650 0.11 (*P*_CO2_ : 0.170 atm)/650	[70]

SDC/Li : Na : K Sym. disc	1200	CO_2_ : H_2_ : N_2_ = 10 : 1 : 10	He	0.26 (*P*_H2_ : 0.21 atm)/650 0.13 (*P*_H2_ : 0.05 atm)/650	[26]

SDC/Li : Na Hollow fibre	100	CO_2_ : N_2_ = 1 : 1 CO_2_ : H_2_ : N_2_ = 10 : 1 : 10	He	5.46/700 (5% H_2_ added as feed) 4.78/700 (no H_2_ added as feed)	[63]

Ag/Li : Na Sym. disk	960	CO_2_ : O_2_ : N_2_ = 3 : 2 : 15	9.41% H_2_-Ar 4.35% H_2_-Ar Ar	1.02/600 0.73/600 0.49/600	[23]

## Data Availability

The data used to support the findings of this study are provided within this article and are available from the corresponding author upon request.
